# The Syracuse Hahnemannian Club

**Published:** 1888-05

**Authors:** Frederick Hooker

**Affiliations:** Secretary


### THE SYRACUSE HAHNEMANNIAN CLUB.

As stated in the last issue of THE HOMŒOPATHIC PHYSICIAN, the "Syracuse Hahnemannian Club" was organized February 24th, 1888, for the purpose of disseminating a "more perfect knowledge of pure Homœopathy as taught by Hahnemann, of the *Organon*, *Materia Medica*, and *Symptomatology*" among its members.

The call for the meeting was signed by the following physicians: E. J. Robinson, Frederick Hooker, R. S. True, J. Willis Candee, A. B. King, Wm. A. Hawley, C. Schumacher, A. J. Brewster, J. W. Sheldon, E. O. Kinne, G. N. Macomber, S. Seward, E. N. Flint, H. D. Emens, F. B. Putnam, John Nottingham.

Dr. S. Seward was elected President, and Dr. Frederick Hooker, Secretary.

It was decided to study the *Organon*, paragraph by paragraph, and to devote as much time as possible to *Materia Medica*, seeking to bring out those practical points in relation to any drug under consideration which had been verified.

At the second meeting, held March 2d, Section 1 of the *Organon* was first discussed, and it was decided that it did not admit of the use of palliatives.

Dr. Brewster remarked that palliative was simply a "covering up" of the symptoms.

The remainder of the evening was devoted to the discussion of Dr. True's paper on Sepia, by Drs. Hawley, Brewster, Seward, Robinson, A. B. Kinne, and Hooker.

Dr. Hawley said he considered itching, aggravated by scratching, as characteristic of Sepia, and that when he has found a case of leucorrhœa with white discharge, staining linen yellow, accompanied by itching of the vulva increased to an intolerable degree by scratching, he has given Sepia with curative effect. Never give Sepia when there is amelioration from scratching.

Dr. Brewster said that the head symptoms are aggravated by motion; ameliorated by continued violent motion.

Dr. Seward said that itching in the bends of the knees, elbows, and on the hands is characteristic of Sepia.

Dr. Kinne said that quarrelsomeness of the Sepia patient differentiated, in the mental sphere, that drug from Pulsatilla.

At the third meeting, held March 9th, Dr. A. J. Brewster gave a clinical talk on Arsenicum.

After giving several clinical cases, in which Ars. in the crude form had produced provings, he gave it as his opinion that remittent fever where the febrile movement increases from noon to midnight and then passes off without sweat, but with great anguish, prostration, and distress, calls for Ars.

Always uses 10M (F.) and sometimes repeats as often as once in twenty-four hours.

Case—A woman eighty years of age, said to have cancer of the tongue, with restlessness, thirst, pain to ear, to temple, better on sitting up, angry swelling of sublingual gland. Ars.$^{10m}$ cured.

Case—Farmer, aged fifty, could not walk, dragged his feet, could not move without exhaustion; distressed look. Ars.$^{10m}$ cured promptly.

Case—Woman, aged thirty, scalp red as beef; fine, white, scaly eruption, gradually spread so that head and face looked like a piece of raw beef; burning, stinging pain. Ars.$^{6x}$ aggr., Ars.$^{10m}$ cured promptly.

Dr. Hawley said he had many times verified this symptom, given by Dr. Gregg, cough with stitching pain at upper part of right lung.

Had one case in which this symptom was present, patient would press hand to spot; confined to bed. Ars.$^{40m}$ (F.) cured at once. Verified again on this patient and on many others.

Dr. Seward said he had almost always repeated.

Case—Child with post-scarlitinal dropsy. Could not hold head up; could not have feet high or she could not breathe; legs had burst; urine two or three tablespoonfuls in twenty-four hours. Ars.$^{18, 12, 6, 3m}$, relief; Ars.$^{cc}$, went to sleep in thirty minutes, and during the next twenty-four hours passed from four pints to six pints of urine.

Dr. Hooker said he had a patient who complained of gnawing pain in stomach, symptoms seemed to call for Sulph., then for Nux, but neither of these relieved; there was great pallor. Ars.$^{3x}$ relieved, and on its return Ars.$^{cc}$ removed it almost instantly.

Gave Ars.$^{cc}$ for same symptoms in a patient who was improving under Sepia with instant relief.

Dr. Schumacher said he had a case of rheumatism in left lower limb, pain from hip down, aggravated by motion. Bry. and Rhus gave no relief. He then complained of a burning pain in the limb, aggravated by uncovering. Ars.$^{30}$, one dose, cured.

Dr. True mentioned several cases of poisoning by Arsenite of Copper which had come under his observation. Before the cause was discovered the patients were supposed to be suffering from a fever. Stools green, involuntary, terrible tenesmus, thirst. Ars. was given and aggravated. The cause was discovered and removed and all got well.

Dr. Hawley spoke of a case of puerperal peritonitis following a protracted difficult labor. Great pallor, abdomen much distended and sensitive, frequent vomiting, thirst for a little and often: metritis. Ars.$^{30}$, in repeated doses, cured.

Dr. Brewster said that Ars. has a headache somewhat like Glon., but with Glon. the brain beats in waves against the skull, while with Ars. the beating seems to be in one spot—a thumping as if it would burst the skull at the point where it strikes.

At the fourth meeting, held March 16th, Sections 2, 3, 4, and 5 of the *Organon* were discussed.

Dr. Hawley said that he found the taking of a case correctly the most difficult thing in the practice of Homœopathy. Hering says, "If you ask leading questions you may conclude that you are yet a novice. Make the patient describe his sensations.

Dr. Candee gave a clinical talk on Apis, stating the following to be his guiding indications:

Aggravation at five P. M.

Drowsiness, dullness of head, starting and shrieking. Suppression of urine (here resembling Cantharis).

Œdema, general dropsy, especially renal. Œdematous swellings, waxy.

Pale redness of throat, œdema of uvula—thirstlessness.

Urticaria, pale, puffy swelling, with stinging pain.

Drs. Seward, Brewster, Sheldon, Hawley, Robinson, and True participated in the discussion which followed.

Dr. Seward said he was once called to see a middle-aged man who complained of a feeling "as though flying through the air" while asleep. Apis cured.

Dr. Hawley said he had used Apis successfully several times, guided by an œdematous swelling of the vulva. Thinks it characteristic.

Case—Woman, aged sixty, chronic headaches, nervous, hystical, œdematous, pale, doughy swelling of the right labium. Apis$^{52m}$ cured.

Have often been led to use Apis by a puffiness under the eyes.

Case—A man was stung on the lip by a honey-bee; immediately tingling of the tongue, sudden very urgent desire for stool, stool flowed away like water, fainting, drenching sweat. Whisky antidoted.

Case—Tonsillitis, tonsil enormously swollen, fluctuation, œdema of uvula, feeling as of a splinter run up to ear. Hepar—no relief. Next morning all symptoms aggravated; pricking as from needles in tonsil, running to ear. Apis$^{52m}$ caused immediate resolution.

Case—Facial erysipelas; Bell. and Rhus failed; stinging as from needles, occiput so sore could not lay head on pillow. Apis$^{52m}$ cured in a few days.

The scream of Apis is peculiar—not *irritable, but agonizing.*

Case—Epithelioma of cervix, burning, stinging pain. Apis$^{52m}$ relieved pain and improved the whole condition.

Dr. Brewster mentioned a case of traumatic erysipelas where gangrene was prevented by Apis.

At the regular meeting of the Syracuse Hahnemannian Club, held March 23d, there were present Drs. Hawley, Candee, Seward, Sheldon, Brewster, Hooker, True, Schumacher, and Robinson.

The meeting was called to order by the President, Dr. Seward.

Paragraphs 6 to 11 inclusive were read.

Dr. Hawley remarked that an attempt to explain many paragraphs of the *Organon* was like an attempt to preach a sermon on the Golden Rule—it only watered it.

Dr. Brewster—There is something unseen that gives form to every particle of matter. What is it? The sick man has the same form, the same features, the same general appearance as when in health. What makes him sick? The science of medicine seeks to find what causes disturbance of health. When there is a deviation of the vital force from the normal standard, the subject will be prostrated by disease unless the tendency is counteracted and the vital force turned into its proper channel.

Dr. Hawley—When a man is sick, he is sick because his vital force is turned out of its normal course. Man *himself* is a form, and the *form* is sick because the vital force is disturbed. Disease is vital force turned out of its normal course by inimical forces.

The Club then proceeded to discuss Rhus tox.

Dr. Candee—Dr. T. F. Allen considers Rhus tox. and Rhus rad. to be identical.

Dr. Hawley—I know there is a difference between Rhus tox. and Rhus rad., for the two plants do not grow alike, and I always cure cases of poisoning by Rhus rad. with one dose of Rhus tox.$^{\text{oc}}$. The emanations from Rhus tox. produce toxical effects.

Dr. Seward—Rhus rad. has stinging and tingling of the tongue, and the red, triangular tip which is imputed to Rhus tox. belong to Rhus rad.

Dr. Robinson—Is not Rhus rad. more superficial in its action than Rhus tox.?

Dr. Hawley—I think that is so, but there is no proof of it.

Dr. Sheldon—I would like to ask if the eruptions of Rhus tox. and Rhus rad. are identical?

Dr. Seward—They are different.

Dr. Sheldon—A child ate one drachm of tablets of Rhus tox.$^{3x}$, and in twenty-four hours she had a fine eruption, which was much like scarlatina; she could keep no covering over her, and scratched the hands and feet constantly. The vesicles were fine, but grew larger as they approached the joints. There was great restlessness and aggravation on keeping quiet.

Dr. Seward—It may be that the itching caused restlessness, instead of muscular and bone pains causing it.

Dr. Hawley—A man was nearly covered all over with eczema, itching, oozing; his shirt would stick to him. Rhus rad.$^{co}$ cured in one week.

Dr. Brewster—An ulcer as large as a silver dollar on the internal malleolus, with itching, stinging, and darting pains. Aggravation *after* motion was cured by Rhus rad. Aggravation *after* motion is characteristic of Rhus rad.

Dr. Seward—If you repeat Rhus tox.$^{cc}$ in antidoting Rhus rad. poisoning you will never cure. *Observe strictly.*

Dr. Sheldon—Would not nature eliminate the poison of Rhus rad. in time?

Dr. Seward—No. Have had one case of eighteen years' standing.

Dr. Robinson—Cured a case of one year's standing with Rhus tox.$^{3x}$.

Dr. Hooker—Saw a case of Rhus rad. poisoning where the patient got the poison on his hands and carried it to his face and genitals, so that his face, hands, and penis were covered with very large vesicles, and the penis enormously swollen.

At the regular meeting, held March 30th, there were present Drs. Hawley, Seward, Schumacher, A. B. Kinne, Macomber, Robinson, and Hooker.

The Secretary read paragraphs 12 and 13 of the *Organon.*

Dr. Hawley—Is disease ever "subject to the manual skill of surgery?"

Dr. Robinson—A foreign body may induce disease.

Dr. Hawley—In such a case, which would cure quickest, the indicated remedy or the knife?

Dr. Macomber—I would combine the two.

Dr. Robinson—In the case of a felon caused by traumatism, the knife alone would cure.

Dr. Seward—When the cause is constitutional, as in the case where a child has necrosis after getting wet, the indicated remedy will cure.

Dr. Hawley—I think we get a quicker and more complete cure without surgical interference in such a case. A child had the entire humerus below the epiphysis resected for necrosis and never got well. A lady had necrosis of the humerus involving the elbow joint, which was anchylosed completely. She was treated by an allopath for fifteen months, and there were at the end of that time four sinuses leading to the necrosed bone. The patient was pallid and emaciated, and could only walk a few feet with assistance.

She was out in a short time after I began to treat her, and now she is strong and well, but there are still three sinuses and occasionally a discharge of spiculæ of bone. I think if I had removed the dead bone I should have had a funeral.

Dr. Kinne—If called to a case of gangrene which started in the toe and extended to above the ankle, where a line of demarcation had formed, what would you do after the indicated remedy failed to relieve?

Dr. Hawley—Amputate. I think Dr. Ad. Lippe put the thing in a nutshell when he said, "To attempt to cure *mechanically* what is caused *dynamically* is an absurdity."

Dr. Hawley then gave a talk on Calc. os. as follows:

Was called to see a woman who had been confined to bed for eighteen months with an ulcer of the right tibia and necrosis of the bone, which had been scraped without benefit, and she had been given up by the allopaths.

Light complexion; sandy hair; blue eyes; cold, damp feet—Calc.-os.$^{6m}$ given first in February and repeated once or twice up to June cured.

Calc. os. has sweating of the head in infants while sleeping and while nursing.

I sometimes give Calc.$^{30}$ once per day for a week and then let it act.

In young ladies, with light blue eyes, who are troubled with falling out of the hair, it will clench the hair so you can hardly pull it out.

Dr. Schumacher—A man, aged forty, had pneumonia three years ago. Constant pain in one spot in the chest. Spot about as large as a dollar; breathing painful. Calc. os. cured.

Dr. True—A woman slipped in getting into a cutter and scraped her shin on the step so that the leg immediately swelled and three large ulcers formed on the front of the leg; great fetor; aggravation from letting the leg hang down. Calc. os. high cured but there is still some œdema of the leg.

Dr. Kinne—I would ask Dr. Hawley for characteristics of the cough.

Dr. Hawley—I am guided by the concomitant symptoms.

Dr. Seward—We do not find the symptom, "cold, damp feet," in the provings.

Dr. Hawley—It is a clinical symptom.

Dr. Seward—I was called to see a case that had been under mongrel treatment for two weeks for pneumonia. Found the woman much emaciated, except her feet and legs to knees, which where œdematous, large, and cold. Expectoration yellow and gray; the gray very fetid; no appetite; tongue thickly coated. She could not lie down, nor rest with her head back in the chair; she had to have her head held by a hand on her forehead. Calc.$^{30}$, one dose, relieved so that by the second day she could lie at an angle of forty-five degrees and on the third day she could sleep quietly and the œdema left the extremities, while on the fifth day she was hungry and ate veal pot-pie, which brought on diarrhœa, which a few doses of China$^{30}$ cured.

At the regular meeting, held April 6th, there were present Drs. Hawley, True, Brewster, and Schumacher.

In the absence of Dr. A. B. Kinne, the regular essayist, Dr. Hawley gave a talk on Psorinum.

Dr. Hawley—Psorinum has dark, watery, stinking stools; they are fearfully offensive; children fret, fret, fret all the time; eruption may be vesicular, but is more apt to be scaly; intense itching; worse at night; relieved by scratching until it bleeds; unclean smell about the person, which no amount of washing will remove.

When other remedies fail to work, though indicated.

Case of eczema behind the ears, on the scalp, and in the bends of the elbows and armpits, accompanied by abscesses affecting the bones.

Nothing relieved, but the eruption disappeared to re-appear again, years after, on the wrists. There was then a patch on each wrist as large as a half dollar, with intense itching, preventing sleep, with constant desire to scratch. Psorinum$^{mm}$ (Swan). In two days the patches had doubled in size and in a week more they had extended to the dorsi of hands between the metacarpal bones, but in another two weeks they disappeared entirely.

In closing this report, it is only justice to remark that the Club owes its existence to the efforts of Dr. R. S. True.

FREDERICK HOOKER, *Secretary*.

